# Generalized radiation model for human migration

**DOI:** 10.1038/s41598-021-02109-1

**Published:** 2021-11-22

**Authors:** Christian Alis, Erika Fille Legara, Christopher Monterola

**Affiliations:** Analytics, Computing and Complex Systems Laboratory (ACCeSs@AIM), Asian Institute of Management, 123 Paseo De Roxas, Makati City, 1229 Philippines

**Keywords:** Applied physics, Statistical physics, thermodynamics and nonlinear dynamics, Mathematics and computing

## Abstract

One of the main problems in the study of human migration is predicting how many people will migrate from one place to another. An important model used for this problem is the radiation model for human migration, which models locations as attractors whose attractiveness is moderated by distance as well as attractiveness of neighboring locations. In the model, the measure used for attractiveness is population which is a proxy for economic opportunities and jobs. However, this may not be valid, for example, in developing countries, and fails to take into account people migrating for non-economic reasons such as quality of life. Here, we extend the radiation model to include the number of amenities (offices, schools, leisure places, etc.) as features aside from population. We find that the generalized radiation model outperforms the radiation model by as much as 10.3% relative improvement in mean absolute percentage error based on actual census data five years apart. The best performing model does not even include population information which suggests that amenities already include the information that we get from population. The generalized radiation model provides a measure of feature importance thus presenting another avenue for investigating the effect of amenities on human migration.

## Introduction

Understanding and predicting the rate of flow between locations have applications in urban and transport planning^1,2^, epidemic modelling^3–6^ and emergency management^7,8^, among others. For many years, the gravity model and its variations^9^ have been the go-to model for predicting these movements. In this model, migration flow is proportional to the population of the source and destination localities, and inversely proportional to their distance.

More recently, the radiation model (RM) for human migration^10^ was introduced and predicts the average flow of migrants $\langle T_{ij}\rangle$ from locality *i* to locality *j* as

1 $$\begin{aligned} \langle T_{ij}\rangle = T_i\frac{p_i p_j}{(p_i+s_{ij})(p_i + p_{j} + s_{ij})} \end{aligned},$$

where $T_i$ is the total number of migrants from *i*, $p_i$ and $p_j$ are the population in *i* and *j*, respectively, and $s_{ij}$ is the total population in the circle centered at *i* and touching *j* excluding the source and the destination populations. It has been shown that this model and its variations can replicate the observed changes in population across several cities in developed countries^2,10–13^ but less so in developing countries^6,14^.

**Figure 1:**
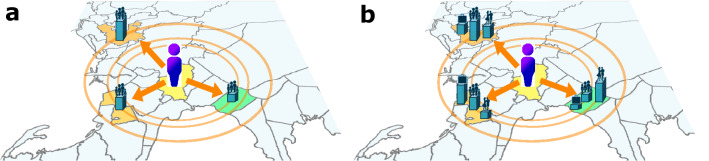
Radiation Model vs Generalized Radiation Model (**a**) In the original Radiation Model, a migrant is pulled more towards a locality if the locality has more economic opportunities as proxied by population, if it is closer to the origin locality, and if there are fewer neighbors with a significant pull. (**b**) In Generalized Radiation Model, the pull due to economic opportunities and the use of population as proxy are no longer required. Instead, features characterizing a locality are used instead, in particular, counts of amenities as well as population and population density. This approach would then be able to capture non-economic motivations for migration, e.g., better quality of life, as well as better estimate economic opportunities e.g., by counting the number of offices. Note that for clarity, only three destination localities are shown here but both original and generalized radiation models look at all non-source localities as a possible destination locality.

The idea behind the model (Fig. 1a) is that migrants are motivated to move towards localities with better economic opportunities such as availability of jobs. However, the pull of one locality is tempered by the pull of neighboring localities as well: a highly urbanized city would have a stronger pull if it is surrounded by rural areas compared to it being part of a metropolis. Similarly, the model gives preference to migration between localities that are nearer to each other over longer distance migrations.

Instead of using actual economic indicators to measure the economic opportunities in a locality, the model uses population as a proxy: the bigger the population of a locality the more economic opportunities it has. However, for developing countries, this assumption may not hold. Due to higher likelihood of inequality in a developing country, a bigger population may not necessarily imply more economic opportunities. In fact, because of increased competition for limited economic opportunities in a crowded city, residents may be tempted to move out to less crowded localities with relatively more opportunities per capita. Moreover, even if the economic opportunities per capita is better, poorly regulated cities in developing countries are challenged by lower quality of life due to crime, pollution, traffic congestion, and weak peer/community support system. For developing countries, religion or faith-related culture can also play a role in one’s migration decision (for example, Muslims are not welcome in some Christian-majority areas and vice versa) as discrimination is often enhanced by poor quality of education^15,16^. The importance of tribal, cultural and linguistic differences has already been shown to affect human mobility in a developing country significantly more than that for a developed country^5,14^.

Even in developed countries, individuals may want to move to a locality in search for a better quality of life instead of better employment^17^. Some migrants do not stay in one city and sometimes even return to where they were before^18^. Indeed, it is already well known that some residents in urban areas opt to move to rural areas following a process collectively known as counterurbanization^19^. Moving to a rural area (usually has lower median income) from an urban area (typically has higher median income) may even result in higher income for the migrant if they move from a lower portion of the income distribution in their original location into a higher portion of the income distribution in the new location^20^. More recently, evidence has been found that the lateral movement of people from one rural area to another is a significant chunk of rural in-migration^21,22^ hence we cannot always assume that people from rural areas will move to urban areas if ever they move.

In this paper, we propose a generalized radiation model (GRM) for human migration. Instead of using population as the only proxy (Fig. 1b), we combine it with other characteristics of the locality to form an urbanization index *U*. We then use *U* for estimating $\langle T_{ij}\rangle$ instead of population:

2 $$\begin{aligned} \langle T_{ij}\rangle = T_i\frac{U_i U_j}{(U_i+v_{ij})(U_i + U_{j} + v_{ij})} \end{aligned},$$

where $U_i$ and $U_j$ are the urbanization index at *i* and *j*, respectively, and $v_{ij}$ is the total urbanization index in the circle centered at *i* and touching *j* excluding source and destination populations.

Aside from improved applicability of GRM to more countries, another benefit of GRM is that it provides another method for investigating the drivers of migration since *U* is composed of several components.

There were already attempts at generalizing the radiation model. Kang et al.^13^ introduced a correction for spatial scale as well as the amount of push from the source, pull towards the destination, and interventions in between. Liu and Yan introduced the opportunity priority selection^23^ and universal opportunity^24^ models that generalize how trip selection by individuals is influenced by the opportunities at destinations and intervening opportunities from source to destination. None of these generalized models, however, directly model how multiple features such as amenities contribute to the attractiveness of opportunities in a place or locality.

Similar to Robinson and Dilkina^25^ which directly estimated $\langle T_{ij}\rangle$ from exogenous data and to McCulloch et al.^26^ which created an ensemble of models, we used machine learning to build the model. However, by anchoring GRM on the radiation model, our model is more mechanistic and easier to interpret compared to a pure machine learning model. By using the amenities in each locality as our feature, we are able to estimate the attractiveness of each amenity type for migrants.

In the next section, we elaborate more on GRM especially on how to estimate *U* and $T_i$. We then explore the fitted GRM for a developing country, the Philippines, and compare it with that of RM.

## Urbanization index

The urbanization index *U* is the analog of population in GRM. It is simply the weighted sum of component factors $f_k$,

3 $$\begin{aligned} U = \sum _k w_k f_k, \end{aligned}$$

where $w_k$ is the weight of the *k*th factor. The factors $f_k$ can be any feature of a locality. Equation (3) is similar to a regression equation and can be extended to model interactions as $w_k f_i f_j$. However, unlike in nonlinear regression, the other side of the equation is not the observed variable but is another variable (attractiveness) that is then used to model the observed variable (population flow). In this paper, we consider the population, population density and the number of structures in the locality for different kinds of amenities as the features $f_k$.

The population density is the census population of each locality over its land area which makes density strongly affected by the polygon definition. In RM, only population is considered but we decided to incorporate population density because it describes the crowdedness of a locality and urban areas tend to be more crowded.

The intuition behind the use of amenities as features is that a migrant may want to move to a locality based on what is important to them according to the culture of the segment of population to which they belong. While the default feature of job opportunities can be represented by the number of offices and/or office space, it also weights independently other factors. For example, presence of schools might be attractive for a family hoping to have the next generation lift them out of poverty. In another case, for relatively well-off families, the presence of considerable leisure places might be more attractive as it suggests a better work-life balance. These various motivations can be readily modeled by the use of amenity counts in a locality, resulting in a more granular and less biased estimation of quality of life. For the standard radiation model, all of such scores are generically simplified to be based on the relative population. Indeed, the importance of amenities or places were already hinted at empirically by Noulas et al.^27^ and is a central concept of Stouffer’s intervening opportunities theory^28^.

The $f_k$’s have varying scales hence the values of each feature should be normalized to make the features comparable to each other. Instead of picking a method of normalization arbitrarily, we investigate four methods of normalization:

- *Min-max* The value of each feature is first rescaled by taking its logarithm owing to the heavily skewed distribution of the values. The transformed values are then further rescaled to [0,1] corresponding to the minimum $x'_\text {min}$ and maximum $x'_\text {max}$ transformed values of the feature,
  4 $$\begin{aligned} x'&= \ln (1 + x) \end{aligned}$$
  5 $$\begin{aligned} x&\rightarrow (x' - x'_\text {min}) / (x'_\text {max} - x'_\text {min}). \end{aligned}$$
  This normalization implies that the magnitude of a feature matters. Thus, if the locality with the maximum value is an outlier then that locality would yield a much stronger pull for migrants while the other localities would have similar pull for that feature.
- *Adjusted* *z-score* The *z*-score of each feature value, after taking its logarithm, is computed but since the transformed value cannot be negative, we translate the value to the right by one standard deviation then set to zero those that are still negative,
  6 $$\begin{aligned} x'&= \ln (1 + x) \end{aligned}$$
  7 $$\begin{aligned} x_\text {adj}&= (x' - \bar{x'}) / \sigma + \sigma \end{aligned}$$
  8 $$\begin{aligned} x&\rightarrow {\left\{ \begin{array}{ll} x_\text {adj} &{} x_\text {adj} \ge 0\\ 0 &{} x_\text {adj} < 0 \end{array}\right. }. \end{aligned}$$
  This normalization also implies that the magnitude of a feature matters. Compared to *Min-max*, there is a stronger bias towards localities that have high values for that feature because those that have low values (more than 1 standard deviation less from the mean) would have zero weights while those with positively outlying values would be more emphasized.
- *Logistic* *z-score* Similar to *Adjusted* z-*score*, the feature value is first transformed to its *z*-score but, instead of translating then thresholding it, it is passed to the standard logistic function to yield a value in [0,1],
  9 $$\begin{aligned} x \rightarrow \frac{1}{1+\exp \left[ -(x - {\bar{x}})/\sigma \right] }. \end{aligned}$$
  Outliers would have transformed values close to 0 or 1 but would not drive it to extreme values which would heavily distort the model.
- *Percentile* The percentile of the value for that feature is used. This implies that migrants only look at the relative rank of the locality and not on the actual value for that amenity. Thus, outliers would not distort the implied pull by that amenity.

Since the features are scaled independently of each other, the superlinearity of some features with respect to population would not matter as much. Their superlinearity would not guarantee that they would have a heavier feature importance compared to other features.

Together with the estimated weights of each feature, the above normalization procedure provides an anchor for interpreting the hierarchy and dynamics of the features used with respect to model accuracy. This will be highlighted in the discussion of results.

Both $T_i$ and $w_k$ can be considered as trainable parameters. The change in population $\Delta p_i$ of locality *i* is

10 $$\begin{aligned} \Delta p_i = p_i^{t+1} - p_i^t = \left( b - d\right) p_i^t + \sum _{r \ne i} \langle T_{ri}\rangle - \sum _{r \ne i} \langle T_{ir}\rangle , \end{aligned}$$

where $p_i^t$ and $p_i^{t+1}$ are the population at times *t* and $t+1$, respectively, for locality *i*, and *b* and *d* are the birth rate and death rate, respectively. To train $T_i$ and $w_k$, we need the population of the localities for two time points to compute $\Delta p_i$. We can then use stochastic gradient descent to minimize the mean squared error (MSE) between the observed $\Delta p_i$ and the estimated $\Delta p_i$. For performance reasons, we expressed $T_i$ as $T_i = \alpha_i p_i$ where $\alpha_i$ is the fraction of the population $p_i$ that are migrants. We then constrained $\alpha_i$ to be between 0 and 1, both exclusive, whilst $w_k$ can be any real number.

The trained $w_k$ and *U* are highly interpretable. A more positive value of $w_k$ implies that $f_k$ drives people to move towards the locality. Similarly, a more positive *U* has more pulling power compared to other neighboring localities.

## GRM in a developing country

We compare the results of GRM using four normalization methods and classic RM for a developing country, the Philippines. We also add a baseline model wherein we scale the local change in population according to the national change in population according to the census. This model implies a uniform birth, death and migration rate, which are equal to the national rates, in all localities.

Census data of the Philippines was taken from the Philippine Statistics Authority website (psa.gov.ph). The three most recent censuses were conducted in 2007, 2010 and 2015, thus, we use the 2007 census as the base year, 2010 census for calibrating the model and 2015 census as the test year. We consider the administrative level 2 (city and municipality) population, which we simply refer to as locality.

We consider all localities in the Philippines as of the base year 2007. This consists of 1627 localities, 136 of which were officially-designated cities based on income level, population and land area. We did not consider metropolitan areas collectively so, for example, the City of Manila and Quezon City were taken separately even though they are both parts of Metro Manila. We also did not restrict the localities to urban areas only because rural areas still pull migrants as pointed out above. Tourism is also a major source of employment in the Philippines and tourist areas are usually rural. For simplicity, we used the Haversine distance between localities even though the Philippines is an archipelago.

**Figure 2:**
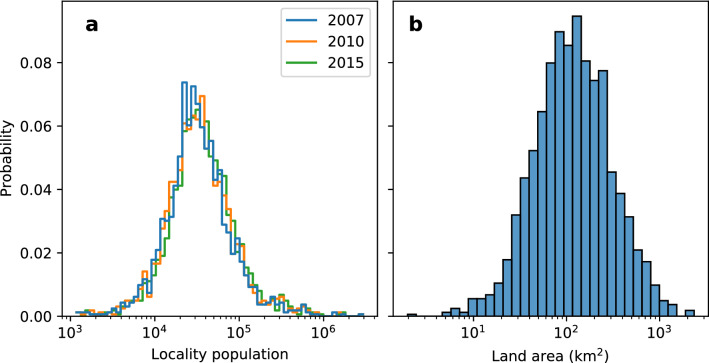
Locality size distributions. The size distribution of the 1627 localities according to (**a**) population and (**b**) land area follow positively skewed distributions with median population size of around 32,000 residents and media land area of about 100 km$^2$.

The distributions of population and land areas of the localities are shown in Fig. 2. These are positively skewed with median population of about 32,000 residents and median land area of about 100 km$^2$.

As typical for many developing countries and even in some developed countries, we are not aware of any available data on migration flows in the Philippines. Hence, we use locality population forecasting performance as the measure of model quality.

Forecasting the population for a year that is after the calibration year (2010) is done by iteratively creating annual forecasts until the desired year is reached. We start by forecasting the 2011 population (calibration year + 1) based on the projected amenity counts for 2010, and the birth and death rates for 2010. Since the trained model is calibrated for a three-year timestep (2007-2010), the raw prediction is divided by three to make it annual. Based on the forecasted population for 2011, we project the amenity counts for 2011, which we then use to forecast the population for 2012 (calibration year + 2) and so on. In all cases, we use the actual birth rate and death rate of the previous year. This information is, of course, not available if we are really forecasting five years into the future but since we are only using the forecasts to compare models, the use of actual rates should be acceptable. The quoted performance metric values in this paper should therefore be considered to be the best possible values of the models.

For comparing the performance of models, we look at the mean absolute percentage error (MAPE) between the forecasted locality population in 2015 with the census population,

11 $$\begin{aligned} MAPE = \frac{1}{N} \sum _i \left| \frac{{\hat{p}}_i^{2015} - p_i^{2015}}{p_i^{2015}} \right| \times 100\%, \end{aligned}$$

where *N* is the number of localities, ${\hat{p}}_i^{2015}$ is the forecasted population in 2015 for locality *i* and $p_i^{2015}$ is the population in 2015 for *i* according to the census. This measure is more relevant than the usual MSE because the distribution of population across localities is fat-tailed so MSE will be heavily biased towards localities with larger population.

We also considered minimizing the forecast MAPE directly as well as minimizing the MSE of the difference in the logarithmic forecast and logarithmic actual population. However, both resulted in worse performance so they are no longer further described in this paper.

Adding more features or predictors in the model would likely improve the performance of the model. However, blindly increasing the number of features would result in overfitting so the forecast performance, which is our basis for ranking model performance, would also suffer.

In order to compute a confidence interval which would be useful for comparing model forecast performance, 100 realizations of the model were trained for every model configuration. The complete table of performance metrics for all the 61 configurations that we investigated is in Supplementary Material Section S1.

## Backcasting the number of amenities

We use OpenStreetMap (OSM) data for counting the number of amenities per locality. Administrative boundaries are courtesy of GADM v3 (gadm.org). Amenity information is based on the available information on OSM on 1 Aug 2015, the first day of the 2015 census, and reconstructed from the 24 Feb 2020 historical OSM data dump.

Although the OSM road coverage for the Philippines is quite high^29^, we are not as confident with points-of-interest (POI) coverage especially in the earlier years 2007 and 2010, corresponding to the base and target census years. To minimize issues of coverage, we instead create a machine learning model to predict the number of each amenity based on the number of residents within 1 km, 5 km to 50 km at increments of 5 km, using census and population data for 2015 as training set. We then use these trained models to predict the amenity counts for 2007 and 2010. We took the logarithm of each amenity type then train the following machine learning models per amenity type: (1) linear regression, (2) support vector machine, (3) gradient boosting method, (4) *k*-nearest neighbors regression, and (5) power law regression. For each amenity type, the trained model that will ultimately be used to predict that amenity type’s count is picked based on test $R^2$. The hyperparameters that were tried for each model are listed in Supplementary Material Section S2.

Many urban indicators do scale with population according to a power law relation^30^, and we could have only used a power law regressor to backcast the amenities for 2007 and 2010. However, by using machine learning, we are able to exploit both linear and nonlinear relationships between population and amenities, which are not fully exploited by using a power law fit. The soundness of this approach is further supported by having power law regression as the selected best model only for 17 (49%) out of 35 amenities, and not 100% as it would be if backcasting using a power law fit is enough. The selected model for each feature is listed in Supplementary Material Table S5.

## Results

**Figure 3:**
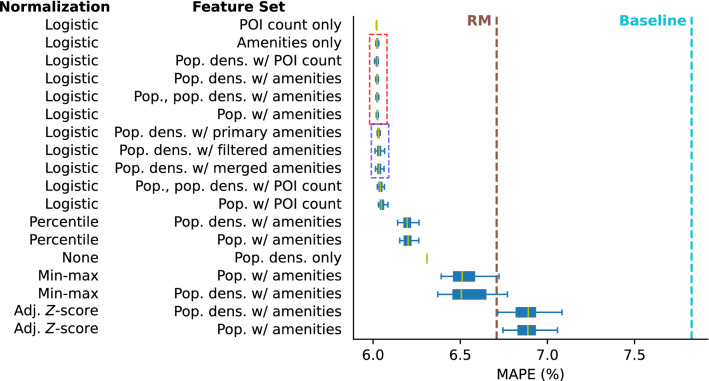
Model performance based on mean absolute percentage error (MAPE). Models in broken-line boxes are not significantly different based on ANOVA. *Logistic* z-*score* normalization is the best method of normalization, outperforming *Percentile*, *Min-max* and *Adjusted* z-*score* normalizations in all instances. The best performing model uses only the number of points of interest in a locality as feature and follows *Logistic* z-*score* normalization. It corresponds to a 10.3% MAPE improvement relative to Radiation Model. All models beat the baseline model which is the outright scaling of locality population according to the same rate of change in the national population. The performance metrics of all of the 61 configurations that were investigated are displayed in Supplementary Table S1.

### Normalization

Figure 3 clearly shows that *Logistic* z-*score* normalization is the best normalization method among the four methods that have been considered. *Logistic* z-*score* normalization implies that the actual amenity count of a locality for a feature is important to would-be migrants but too many instances of an amenity would eventually saturate. When picking a locality among a set of candidates based on an amenity, a would-be migrant prefers more instances of that amenity but localities that have a lot of that amenity are practically the same. This behavior has been hinted before by Noulas et al.^27^.

*Logistic* z-*score* normalization also facilitates the use of amenity counts, which generally follow a heavy-tailed and heavily skewed distribution. Being bounded from zero to one, the weights generated from *Logistic* z-*score* normalization are readily interpretable and comparable across models.

*Percentile* normalization performs better than the other normalization methods except for *Logistic* z-*score* normalization. Similar to the latter wherein differences in the large value amenity counts do not matter, the former does not consider the actual counts at all but only the relative rank. Having these two normalization methods as best-performing suggests that there is some form of estimation or fuzziness when would-be migrants evaluate a locality for an amenity–something that aligns with what we observe anecdotally.

*Min-max* normalization also performs better than RM and is bounded from zero to one so the weights are readily interpretable and comparable across models. However, for some instances, it performs worse than RM.

The *Adjusted* z-*score* models performed worst, even worse than RM. It is also more difficult to compare and interpret because it is only bounded to the left by 0 but is unbounded to the right.

Due to the consistent superior performance of *Logistic* z-*score* normalization, succeeding results and discussion will focus on models using this normalization method. A more detailed investigation of features were performed only on these models as well.

### Feature sets

Population density seems to be a better feature compared to population. Switching RM to use population density (No normalization, Pop. dens. only in Fig. 3) instead of population results in a 5.9% relative improvement in mean MAPE. Looking at Fig. 3, we see that models with population density consistently outperform those with population albeit by a very small amount (0.0093% to 5.9% relative improvement in mean MAPE among the models in Fig. 3) in many cases.

Combining population, population density and amenity counts (mean MAPE = 6.024097%) does not result in the best model either. However, there is a caveat that its MAPE distribution is not significantly different (One-way ANOVA $F=0.386$, *p*=0.819) from the distributions of four other models (Amenities only mean MAPE = 6.023319%; Population density w/ POI count mean MAPE = 6.023345%; Population density w/ amenities mean MAPE = 6.023551%; Population w/ amenities mean MAPE = 6.024110%).

The number of points of interest (POI), or the sum of amenity counts, is weakly correlated to population (Spearman *r* = 0.3) and this makes sense because urban areas tend to have more POIs than less urban areas. Thus, incorporating amenity or POI information could improve RM while also being used as a measure of attractiveness.

**Figure 4:**
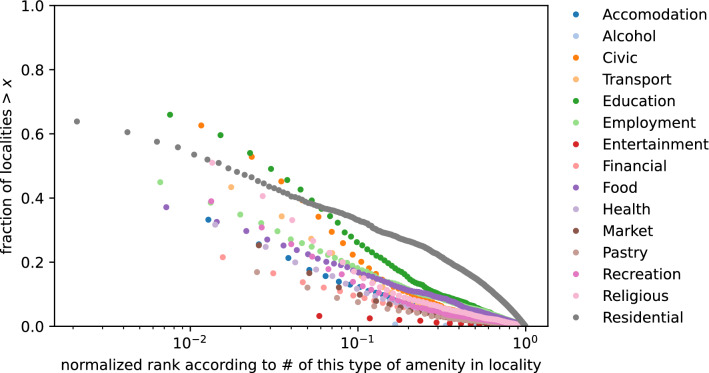
Amenity distributions. The distributions do not collapse to a single distribution which suggests that a single measure e.g., POI count, cannot reproduce the distribution of all the amenities.

In terms of mean MAPE, the number of POIs in a locality as the only feature achieves the best result with a mean MAPE of 6.019906% and 10.3% relative improvement in mean MAPE from RM. However, as shown in Fig. 4, the distributions of the number of each amenity type per locality do not collapse to a single distribution, which suggests that the number of POIs is not a direct representation of amenities. If we want to understand the relative importance or attractiveness of different amenity types then we could use models with amenity types as features which would entail a 0.06-0.07% relative increase in mean MAPE.

To remove redundancy and irrelevant variables thereby possibly improving the accuracy of the predictions as well as the interpretability of the model and weights, we investigate reducing the number of amenities considered in the model by grouping them. Each OSM feature belongs to a primary group^31^ such as building, landuse and shop. Another approach for grouping features is by performing Ward’s agglomerative clustering^32^ of the features based on the locality values. Details of this clustering is found in Supplementary Material Section S3. With these feature groupings, we investigate three methods of reducing the amenity-related features: (1) aggregating amenity count by primary features (*primary amenities*), (2) aggregating amenity count by groups based on feature clustering (*merged amenities*) and (3) selecting a representative amenity for each group based on feature clustering (*filtered amenities*). Reducing the number of amenity-related features results in worse performance compared to the best model but is still better than RM. Among the three methods considered, aggregating amenity counts by primary group has the best performance (mean MAPE = 6.031234%) whilst aggregating amenity counts by feature clustering performed worst (MAPE = 6.042972%). However, one-way ANOVA suggests that the MAPE distributions are not significantly different (*F*=2.846, *p*=0.0596).

The best-performing models are the ones that only uses POI or amenity counts implying that population information is causally related to amenities and hence, by virtue of granularity, captures not just the mean field but also the variability, thereby providing a better model for human mobility.

### Feature importance

**Figure 5:**
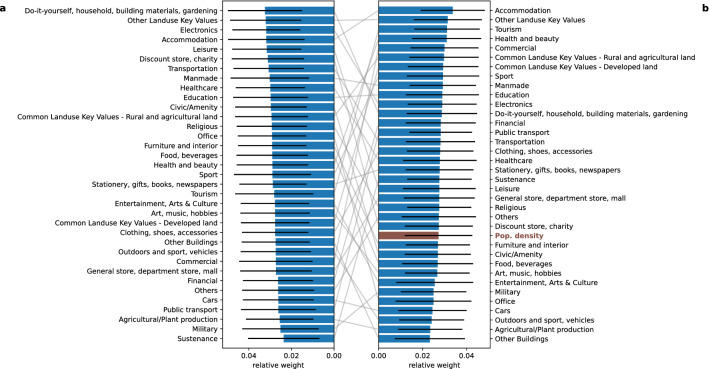
Feature importance of the two best performing models with amenity features. (**a**) *Logistic* z-*score* normalization, amenities only (**b**) *Logistic* z-*score* normalization, population density with amenities. The most important features are not directly related to job opportunities which suggests people move not just because of job opportunities. Population feature importance is not ranked highly, even omitted in the best performing model which suggests amenities already include information derived from population.

We now investigate the relative weights of features e.g., amenity type and population density by looking at the best performing models with amenity features (Amenities only) and population density (Population density with amenities). Their MAPE distributions are not significantly different (*t*-test *t*=-0.472, *p*=0.638). However, for all weights in all models, the *t*-test for determining whether a feature weight has a mean value of 0 yields a *p*-value $\ll 10^{-3}$.

With 100 model realizations (Fig. 5), the standard deviation of the relative weights is quite high which makes the distributions of relative weights of a pair of features not being significantly different from each other for most pairs of features. We therefore look at general trends instead.

The relative weights for the amenities-only model are shown in Fig. 5a. At the outset, the top features do not seem to be related to jobs or work, supporting the initial assertion that migrants may not be solely looking at job opportunities when deciding to move; they may look at other concerns e.g., quality of life as well. However, this observation needs further investigation since more leisure and amenity places correspond to more service sector jobs. The presence of the top features may also correspond to a locality resting in an urban setting which could, in turn, imply more jobs.

When population density is added as a feature (Population density with amenities, Fig. 5b), Leisure seemed to swap with Tourism. Indeed, the top features appear to be related to tourism, which in 2015 contributed 8.2% to the Philippine GDP and 12.7% to total employment^33^. Population density is only the 27th out of 36 features in terms of relative weight. Since tourist areas in the Philippines are usually less urban with less population and population density, relying on just population information would not be able to capture the pull of tourist areas to would-be migrants.

## Discussion

Predicting from and to where people move, and by how much, is one of the fundamental problems in the study of human mobility. RM provides a useful model for human mobility that allows us to answer this fundamental problem.

We extend this model by allowing amenities to be proxies for the migration attractiveness of a locality instead of population alone as in the original model. The model complements an earlier work demonstrating that amenities predict accurately the daily movement of people^34^. The result of the formulation shown here is consistent with how daily unchanging routines eventually accumulates to years resulting in permanent migration in some portion of the population. The model carries with it a natural way of interpreting the driver of migration to the level of amenities not possible in RM. Moreover it allows actionable insights that take into account the sensibilities or cultural preferences of the citizens of a country.

Our work is extensive: we considered different (1) methods of normalization, (2) feature sets, (3) optimization target and even (4) performance metric–distilling the results to only elaborate on the better performing configurations in this paper. It also provides an example of how machine learning can help resolve seemingly circular problems. In particular, using population to estimate amenity counts which will then be used for predicting population seems circular. However, by using machine learning, we were able to break this circular problem by incorporating more complicated forms of nonlinearities, even those that are not invertible, that are not included in the power law model, which is the best theoretical model for the relationship between population and amenities. This approach also resulted in improved prediction, beating power law model 51% of the time.

Our results show that our model outperforms RM outright with as much as 10.3% relative improvement in mean MAPE for the best performing model. GRM beats the baseline and RM models even amidst a likely incomplete OSM data so a more complete OSM data will only improve the accuracy of GRM. Furthermore, amenity features outrank population features in importance with the best performing model not using any population feature at all. This suggests amenities already include information derived from population, which may be simply due to amenities being correlated with population or may actually be a proxy to how people decide to move to a locality based on the amenities in it.

Indeed, amenities out-weighting population information in terms of feature importance offers a couple of applications. The first application is that this can potentially be used for doing population counts (census) in an area. Second, with the feature weights as a guide, we can potentially investigate causality of amenities i.e., by how much people will be attracted to move to a place if we put up a particular amenity there. Of course, by doing so, we would be able to answer the conundrum of whether putting a particular amenity drives people to move there or is it the other way around–an amenity is put up because there are people there.

Generalizing and allowing a better resolved RM is a step closer in understanding more accurately the science of the emergence of cities. While the organization of amenities have been previously presented based on opposing concepts of diffusion and aggregation^35,36^, the complexity of the drivers that balance the built up and growth of cities are still an open concern^37^. The work here provides a procedure for quantifying a critical component of the formation of cities which is the movement of people as a function of the diversity and quantity of amenities.

## Supplementary Information

Supplementary Information.

## Data Availability

All source data are publicly available at OSM (openstreetmap.org), GADM (gadm.org) and Philippine Statistical Authority (psa.gov.ph). Population, population density and backcasted amenity counts are available as DOI 10.6084/m9.figshare.16620562.
