# Supplementary material for: Generalized radiation model for human migration
Source: Sci Rep. 2021 Nov 22;11:22707. doi: 10.1038/s41598-021-02109-1 (PMC8609035; doi:10.1038/s41598-021-02109-1)
Supplement: Supplementary file 1 — Supplementary Information. [file 41598_2021_2109_MOESM1_ESM.pdf]

Generalized Radiation Model for Human Migration

Christian M. Alis (calis@aim.edu)
Erika Fille Legara (elegara@aim.edu)
Christopher Monterola (cmonterola@aim.edu)

Section S1. Performance of all investigated models

Table S1: Mean performance of models after 100 realizations. Optimization target: Δp_i MSE

Normalization	Feature set	MAPE (%)	MAE	MSE
Logistic z-score	POI count only	6.020	8.241 $\times 10^9$	325076
	Amenities only	6.023	8.285 $\times 10^9$	326259
	Pop. dens. w/ POI count	6.023	8.237 $\times 10^9$	325281
	Pop. dens. w/ amenities	6.024	8.278 $\times 10^9$	326201
	Pop., pop. dens. w/ amenities	6.024	8.281 $\times 10^9$	326261
	Pop. w/ amenities	6.024	8.287 $\times 10^9$	326306
	Pop. dens. w/ primary amenities	6.031	8.291 $\times 10^9$	326586
	Pop. dens. w/ filtered amenities	6.043	8.303 $\times 10^9$	326804
	Pop. dens. w/ merged amenities	6.043	8.296 $\times 10^9$	326849
	Pop., pop. dens. w/ POI count	6.052	8.437 $\times 10^9$	328381
	Pop. w/ POI count	6.058	8.415 $\times 10^9$	328628
Percentile	Amenities only	6.196	8.472 $\times 10^9$	334579
	Pop. dens. w/ amenities	6.198	8.466 $\times 10^9$	334512
	Pop. w/ amenities	6.202	8.478 $\times 10^9$	334837
	Pop., pop. dens. w/ amenities	6.204	8.469 $\times 10^9$	334723
	Pop. dens. w/ filtered amenities	6.240	8.417 $\times 10^9$	335386
None	Pop. dens. only	6.310	8.255 $\times 10^9$	340342
Percentile	Pop. dens. w/ merged amenities	6.387	8.439 $\times 10^9$	339635
	Pop. dens. w/ primary amenities	6.393	8.511 $\times 10^9$	340120
Min-max	Pop. w/ amenities	6.538	9.502 $\times 10^9$	353377
	Pop. dens. w/ amenities	6.540	9.493 $\times 10^9$	353343
None	Population only	6.709	9.530 $\times 10^9$	369023
Adj. z-score	Pop. dens. w/ amenities	6.881	1.075 $\times 10^{10}$	380554
	Pop. w/ amenities	6.885	1.062 $\times 10^{10}$	379161
Percentile	POI count only	6.985	8.595 $\times 10^9$	355252

Table S2: Mean performance of models after 100 realizations. Optimization target: log p_i MSE

Normalization	Feature set	MAPE (%)	MAE	MSE
Logistic z-score	Amenities only	6.250	8.102 $\times 10^9$	328726
	Pop. dens. w/ amenities	6.250	8.097 $\times 10^9$	328694
	Pop. w/ amenities	6.251	8.108 $\times 10^9$	328828
	Pop., pop. dens. w/ amenities	6.251	8.103 $\times 10^9$	328782
	Pop. dens. w/ primary amenities	6.259	8.116 $\times 10^9$	329134
	Pop. dens. w/ merged amenities	6.269	8.140 $\times 10^9$	329440
	Pop. dens. w/ filtered amenities	6.271	8.146 $\times 10^9$	329449
Percentile	Amenities only	6.434	8.343 $\times 10^9$	337519
	Pop. dens. w/ amenities	6.435	8.331 $\times 10^9$	337365
	Pop., pop. dens. w/ amenities	6.444	8.338 $\times 10^9$	337642
	Pop. w/ amenities	6.451	8.354 $\times 10^9$	338116
	Pop. dens. w/ filtered amenities	6.531	8.341 $\times 10^9$	340271
	Pop. dens. w/ merged amenities	6.665	8.355 $\times 10^9$	344009
	Pop. dens. w/ primary amenities	6.677	8.426 $\times 10^9$	344605
Min-max	Pop. w/ amenities	6.824	9.136 $\times 10^9$	357355
	Pop. dens. w/ amenities	6.827	9.162 $\times 10^9$	357721
Adj. z-score	Pop. dens. w/ amenities	7.134	1.025 $\times 10^{10}$	382855
	Pop. w/ amenities	7.145	1.012 $\times 10^{10}$	381545

Table S3: Mean performance of models after 100 realizations. Optimization target: p_i MSE

Normalization	Feature set	MAPE (%)	MAE	MSE
Logistic z-score	Pop. dens. w/ amenities	7.001	9.781 $\times 10^9$	371325
	Pop., pop. dens. w/ amenities	7.026	9.817 $\times 10^9$	372067
	Pop. dens. w/ primary amenities	7.052	9.855 $\times 10^9$	372884
	Amenities only	7.057	9.855 $\times 10^9$	372766
	Pop. w/ amenities	7.060	9.861 $\times 10^9$	372947
	Pop. dens. w/ filtered amenities	7.089	9.848 $\times 10^9$	374004
	Pop. dens. w/ merged amenities	7.141	9.928 $\times 10^9$	375585
Percentile	Pop., pop. dens. w/ amenities	7.226	1.001 $\times 10^{10}$	382291
	Pop. dens. w/ amenities	7.250	1.004 $\times 10^{10}$	383142
	Amenities only	7.270	1.008 $\times 10^{10}$	383833
	Pop. w/ amenities	7.290	1.009 $\times 10^{10}$	384290
	Pop. dens. w/ filtered amenities	7.325	1.001 $\times 10^{10}$	384064
	Pop. dens. w/ primary amenities	7.365	1.002 $\times 10^{10}$	385595
	Pop. dens. w/ merged amenities	7.392	9.992 $\times 10^9$	385679
Min-max	Pop. dens. w/ amenities	7.472	1.150 $\times 10^{10}$	398014
	Pop. w/ amenities	7.501	1.156 $\times 10^{10}$	399197
Adj. z-score	Pop. dens. w/ amenities	7.933	1.374 $\times 10^{10}$	433692
	Pop. w/ amenities	8.065	1.388 $\times 10^{10}$	438788

Section S2. Backcasting amenity counts

Table S4: Model hyperparameters

Model	Parameters searched
Gradient boosting model	Loss: Least squares, Huber
	Learning rate: 0.1, 0.01, 0.001
	Number of trees: 100, 1000, 10000
k-nearest neighbors	k: 5, 10, 20
	Distance metric: Euclidean, Manhattan
Linear regression	No hyperparameter
Power law regression	No hyperparameter
Support vector machine	C: 0.1, 1, 10

Table S5: Identified best model for backcasting each amenity type

Amenity type	Best model
Accommodation	Power law regression
Agricultural/Plant production	k-nearest neighbors
Art, music, hobbies	k-nearest neighbors
Cars	Power law regression
Civic/Amenity	Power law regression
Clothing, shoes, accessories	Power law regression
Commercial	Gradient boosting model
Common Landuse Key Values - Developed land	Gradient boosting model
Common Landuse Key Values - Rural and agricultural land	k-nearest neighbors
Discount store, charity	Power law regression
Do-it-yourself, household, building materials, gardening	Power law regression
Education	Power law regression
Electronics	Power law regression
Entertainment, Arts & Culture	k-nearest neighbors
Financial	Gradient boosting model
Food, beverages	Power law regression
Furniture and interior	Power law regression
General store, department store, mall	Gradient boosting model
Health and beauty	Gradient boosting model
Healthcare	Power law regression
Leisure	Power law regression
Manmade	Power law regression
Military	Power law regression
Office	Gradient boosting model
Other Amenities	k-nearest neighbors
Other Buildings	k-nearest neighbors
Other Landuse Key Values	Power law regression
Outdoors and sport, vehicles	Gradient boosting model
Public transport	k-nearest neighbors
Sport	Gradient boosting model
Stationery, gifts, books, newspapers	Power law regression
Storage	Linear regression
Sustenance	k-nearest neighbors
Tourism	Gradient boosting model
Transportation	Power law regression

Section S3. Grouping amenities by agglomerative clustering

We looked at grouping features to reduce the number of dimensions. OpenStreetMap has a suggested hierarchy of amenity types and we can use the primary group of each amenity type for grouping them. Another approach is to derive the groupings by clustering and we did so by performing Ward's clustering on the correlation matrix of the amenity types. A plot of the correlation matrix of the amenity types is shown in Figure S1 and the resulting groupings with the representative of each group is listed in Table S6.

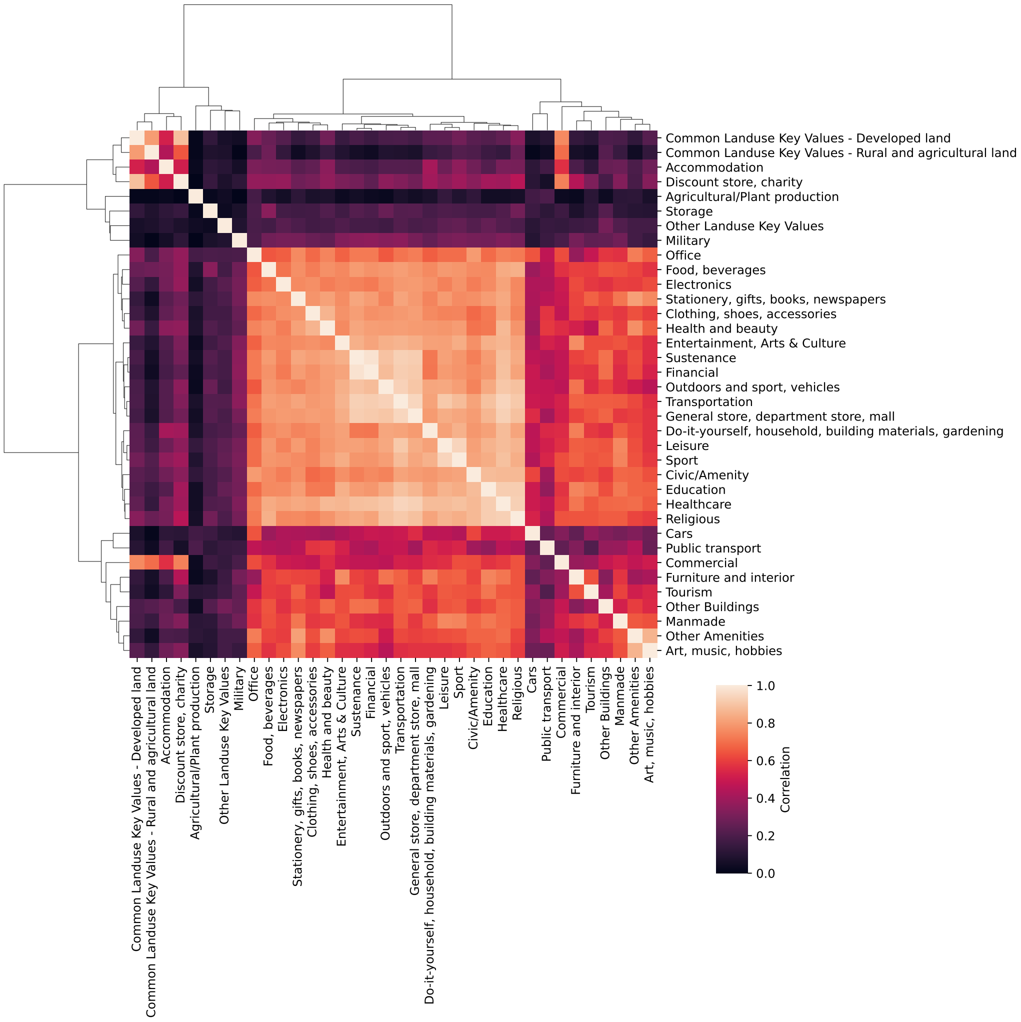

Figure S1: Amenity type correlation matrix. The blocks in the correlation matrix as well as the results of Ward's clustering were used as basis for grouping amenity types.

Table S6: Grouping of amenity types with each representative in bold

Civic/Amenity
Clothing, shoes, accessories
Do-it-yourself, household, building materials, gardening
Education
Electronics
Entertainment, Arts & Culture
Financial
Food, beverages
General store, department store, mall
Health and beauty
Healthcare
Leisure
Office
Outdoors and sport, vehicles
Religious
Sport
Stationery, gifts, books, newspapers
**Sustenance**
Transportation
**Commercial**
Art, music, hobbies
Furniture and interior
Manmade
Other Amenities
Other Buildings
Tourism
**Accommodation**
Common Landuse Key Values - Developed land
Common Landuse Key Values - Rural and agricultural land
Discount store, charity
**Agricultural/Plant production**
**Cars**
**Military**
**Public transport**
**Other Landuse Key Values**
**Storage**
